# Word skipping as an indicator of individual reading style during literary reading

**DOI:** 10.16910/jemr.13.3.2

**Published:** 2020-02-27

**Authors:** Myrthe Faber, Marloes Mak, Roel M. Willems

**Affiliations:** Donders Centre for Cognitive Neuroimaging, Radboudumc, The Netherlands; Centre for Language Studies, Radboud University, The Netherlands; Donders Institute for Brain, Cognition and Behaviour, Radboud University, The Netherlands

**Keywords:** Eye movements, gaze durations, word skipping, narratives, literary reading, individual differences

## Abstract

Decades of research have established that the content of language (e.g. lexical characteristics of words) predicts eye movements during reading. Here we investigate whether there exist individual differences in ‘stable’ eye movement patterns during narrative reading. We computed Euclidean distances from correlations between gaze durations time courses (word level) across 102 participants who each read three literary narratives in Dutch. The resulting distance matrices were compared between narratives using a Mantel test. The results show that correlations between the scaling matrices of different narratives are relatively weak (r ≤ .11) when missing data points are ignored. However, when including these data points as zero durations (i.e. skipped words), we found significant correlations between stories (r > .51). Word skipping was significantly positively associated with print exposure but not with self-rated attention and story-world absorption, suggesting that more experienced readers are more likely to skip words, and do so in a comparable fashion. We interpret this finding as suggesting that word skipping might be a stable individual eye movement pattern.

## Introduction

What drives readers’ eye movements during narrative reading? An important and obvious factor is the content of the language being read. Indeed, decades of research have established that eye movements during reading vary as a function of the content of language. For instance, longer reading times are associated with longer and unfamiliar words compared to their shorter, high-frequency counterparts (e.g., 1, 2, 3, 4). A naïve prediction from these findings would be that all readers show similar eye movement patterns during reading. This would be the case if language content is the *sole* factor driving eye movements, affecting all readers in the same manner. However, this is unlikely, as the alignment between reading times and complexity (cognitive coupling) varies among readers and is predictive of text comprehension (5, 6). Indeed, previous research has shown that differences in reading strategies exist that are reflected in eye movements (e.g., 7).

These findings resonate with previous work in film comprehension. It has been found that individual differences in viewing behavior underlie differences in narrative comprehension. Indeed, small differences in eye movements are significantly linked to the viewers’ mental model of the narrative (8). However, in the context of narrative films, these eye movement differences are very small, as the vast majority of gaze is driven by what is known as the “tyranny of film”: strong constraints on the allocation of visual attention imposed by film editing techniques (8, 9).

In analogy with the “tyranny of film”, the content of a narrative text similarly poses constraints on reading behavior, but it is likely that there is considerable variability given research suggesting that the strength of the constraints might vary between individuals. For instance, reading behavior in readers with higher vocabulary scores (10), better reading skills (11), and more print exposure (12, 13) is less strongly influenced by word frequency. These differences might arise because word recognition might be more automatic in skilled readers compared to less skilled readers (14). For instance, skilled readers skip about 25-33% of the words in a text, with highly frequent two-letter words being skipped more than 75% of the time (14, 15). Words longer than eight letters are unlikely to be skipped, irrespective of skill level (15). In addition, poor readers tend to make longer fixations, in particular in the context of low frequency words (11, 14, 16). Taken together, these results suggest that reading styles might differ across individuals.

However, it is currently unknown which eye movements are stable within an individual, irrespective of the text, and are therefore good markers of individual reading style. In this study, we aim to establish indicators of stable individual reading style differences during literary reading. We investigate two key candidates that could be markers of individual reading styles during literary reading. On the one hand it is possible that readers differ consistently in the amount of time spent on each word (gaze duration). For instance, if less skilled readers’ gaze durations are more influenced by low-level features such as lexical frequency and word length, we expect this to be the case across different texts. A second potential candidate for a stable individual difference in reading style is word skipping, as described above. In this exploratory re-analysis of previously collected eye movement data we investigate stable differences in individual reading styles by correlating each individual’s word-level gaze duration time series with each other individual’s. Next, we compute a Euclidean distance matrix that comprises the differences between all participants in terms of gaze duration patterns. If stable individual reading styles exist, then we would expect that differences between participants would be similar across narratives. We particularly ask whether it is gaze duration per se, or the skipping pattern during reading which is a better indicator—if any exists—of reading style.

## Methods

### Participants

We used an existing sample of 102 participants (81 females) who were recruited from the participant pool of the Radboud University (The Netherlands). This sample has previously been described in (17). All participants were native speakers of Dutch and had normal or corrected-to-normal vision. Participants were on average 23 years old (range 18-40). They received monetary compensation (15 euros) or course credit for their participation.

### Materials

Each participant read three existing literary short stories in Dutch. A full description of the stimuli and apparatus can be found in (17). In brief, these stories were written by acclaimed writers (two contemporary Dutch writers: Van Essen (18) and Van Hassel (19), and one by Nabokov (20), professional, published translation). Stories were 2988, 2659, and 2143 words and took 10-15 minutes to read. Stories were presented in counterbalanced order, and none of the participants reported being familiar with any of the stories.

Eye movement data were collected using a desktop-mounted EyeLink1000Plus system, at a sampling rate of 500 Hz (monocular sampling of the dominant eye when possible; non-dominant eye tracked for seven participants). A chin rest was used to minimize head movements, maintaining a distance of 108 cm between the participants’ eyes and the bottom of the screen. Stimuli were presented using SR Research Experiment Builder software. The experiment was presented on a BenQ XL 20420T 24” LED screen at a resolution of 1024 x 768 (32 bits per pixel). The stories were presented in sections that adhered to the original division into paragraphs as much as possible, resulting in 30 sections per story. Each section was presented with minimum margins of 120 pixels on all sides. The black 15-point Calisto MT font was used, with a line spacing of 24 mm. Areas of interest were automatically defined by Experiment Builder, with boundaries centered between horizontally and vertically adjacent words (no space between areas of interest).

As discussed above, previous work has suggested that skill level might influence reading patterns. We therefore used the Author Recognition Test to assess participants’ print exposure (an implicit measure of reading experience) (21). A Dutch adaptation of this task (22) consists of 42 of names of which participants have to indicate which names are familiar as names of writers (30 real authors, 12 foils). This task (21, 23, 24) and its Dutch adaptation (22, 25) have previously been validated. Participants were instructed to underline only the names they recognize with certainty. The full list of names used can be found in (22). The mean ART score in our sample was 7.32 (*SD* = 4.69; range = 1 – 23).

Reading patterns are also known to be influenced by state- rather than trait-based reader characteristics such as attention to the text (e.g., 26) and absorption in the story (27). We used the Story World Absorption Scale (28) to assess participants’ absorption and self-rated attention to each story. Absorption is typically defined along the dimensions of attention, transportation, emotional engagement, and mental imagery, all of which contribute to the experience of absorption (see (28) for validation of the scale). The original questionnaire consists of 18 items. However, for the purpose of the original study (17), six additional items about perceptual simulation were added (24 questions in total). The questionnaire used here consists of the following subscales and questions: Attention: five items, Cronbach’s *α* = .90; Transportation: five items, Cronbach’s *α* = .87; Emotional Engagement: six items [five original scale, one additional question], Cronbach’s *α* = .90; Mental Imagery: eight items [three original scale, five additional questions], Cronbach’s *α* = .91), which all showed good or excellent reliability in our dataset. An overview of all items can be found in (17). Each item was rated on a 7-point scale ranging from 1 (disagree) to 7 (agree). Here, we focus on the overall SWAS score as a measure of absorption, and on the Attention subscale as a measure of self-rated attention. The mean overall SWAS score was 4.28 (*SD* = 1.07; range = 1.25 – 6.71). The mean Attention subscale score was 4.48 (*SD* = 1.24; range = 1.20 – 7).

### Procedure

Details on the procedure can be found in (17). In brief, participants were instructed to move as little as possible, while reading as naturally as possible. Participants’ dominant eye was identified using an eye dominance test. The experiment took place in a sound proof booth. Reading was self-paced, and there was no time restriction. In between stories and at the end of the experiment, participants filled out several questionnaires pertaining to their reading experience, as we just described above. The questionnaires were the Story-World Absorption Scale (SWAS, for each story; 28) and the Author Recognition Test (at the end of the experiment; 21, 22) (see (17) for details) outside of the testing booth. Each story was preceded by a 9-point calibration and validation session. Drift correction took place every five sections.

### Data Preprocessing

As described in (17) fixations were checked manually and aligned if necessary using SR Research EyeLink Data Viewer before data analysis. If fixations for a section could not be aligned, data for that section were rejected for that participant (2.26% of the total data, which indicates that overall data quality was good). If more than six sections of a story had to be rejected (> 20% data), then data for that story were excluded for that participant (one story for four participants; details on the exclusions can be found in (17)). No sections were rejected for 62 participants. For 40 participants, data for at least one section was rejected (1-6 sections rejected for Story 1: 9 participants, 1.56 sections on average; Story 2: 14 participants 2.14 sections on average; Story 3: 21 participants, 2.05 sections on average).

In our analysis we use gaze duration as the dependent variable. We used gaze durations for several reasons. Firstly, gaze duration is a theoretically meaningful gaze behavior that is linked to the cognitive (e.g., lexical) processing of words (see for instance (6)). Secondly, our empirical question requires a comparison of the eye movements over time across participants (i.e. how dissimilar is reading behavior across participants). To do so, we need to aggregate our data to a level that is common across participants. Our focus on gaze durations at the word level is theoretically meaningful, as it allows us to identify word skipping as well as variability in word reading.

Since we perform correlation analyses on the whole time series, it is necessary to obtain word-level series of gaze durations that are equally long across participants. For our primary analyses, we used data from 102 participants, and coded missing data as NaNs or 0-durations (explained below). In our more stringent analysis, we only include data from the 62 participants for whom none of the sections were removed.

## Results

**Figure 1.**
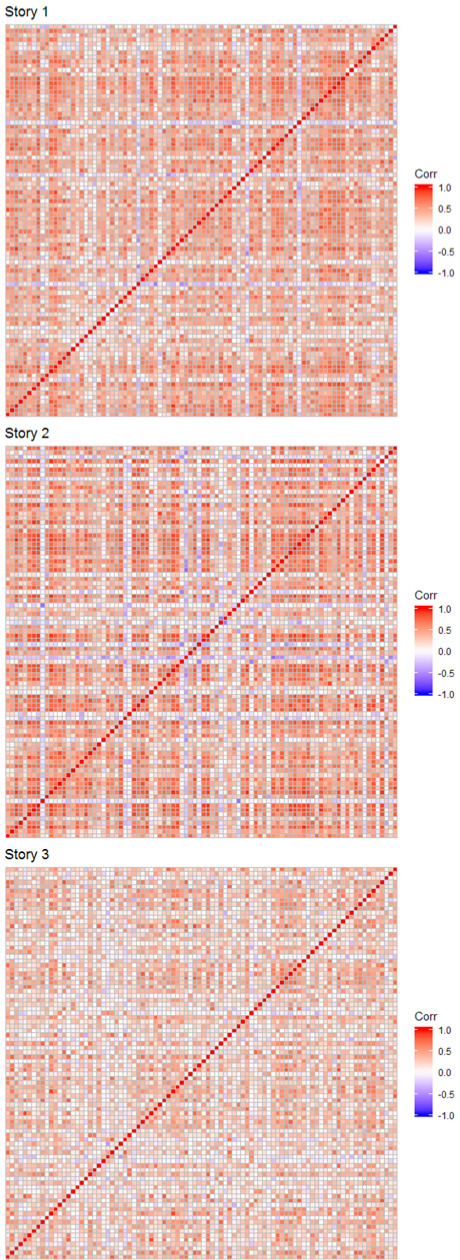
Correlation matrices for each story. Cells represent correlations between all possible subject pairs. The figure presents that correlation matrix when skipped words values were excluded. The matrices show that there is considerable spread in how (dis)similar participants are in their gaze duration pattern. The diagonal of the correlation matrix represents the correlation of each participant with themselves (correlation *r* = 1.00). Red means positive, blue negative correlations.

We first computed the word-level time series of gaze durations for each participant for each story (time series of aggregated durations of all fixations on each word). These time series are equally long across participants (necessary for correlations) and are aggregated to a theoretically meaningful level (the single word, which allows us to identify skipped words). Missing data points (i.e. no gaze duration recorded for a word) were treated in two ways: these data points were either ignored in the analysis, or they were given a 0-second duration to account for words being skipped. These analyses are reported separately below. We then computed correlations between these times series across all participants (i.e. pairwise correlations), for each story separately. In the first approach, we computed correlations based on complete data only (i.e. for each pair of participants, we analyzed only words for which gaze durations were present for both participants). In the second approach, correlations were computed on all data, with 0-duration for words for which there was no gaze observed. Subject-by-subject correlation matrices for each story can be found in Fig. 1 (excluding missing data points).

The between-subject correlations were transformed to Euclidean distances (*d* = √(2(1-*r*))). These distances represent how dissimilar a participant’s reading behavior is relative to each other participant’s reading behavior. We reasoned that if there are stable, individual-level reading behaviors across stories, then the (dis)similarities across participants should be similar across stories. To test this, we conducted a Mantel test on the Euclidean distance matrices for each pair of stories (only participants included whose data is available for that pair of stories), which tests the correlation between two matrices (package “ade4” version 1.7.13 in R; 29). The output of this test is a correlation coefficient and its statistical significance, computed via permutation testing (1000 times using a Monte-Carlo method). A significant correlation suggests that there might be stable, individual-level reading styles.

**Figure 2.**
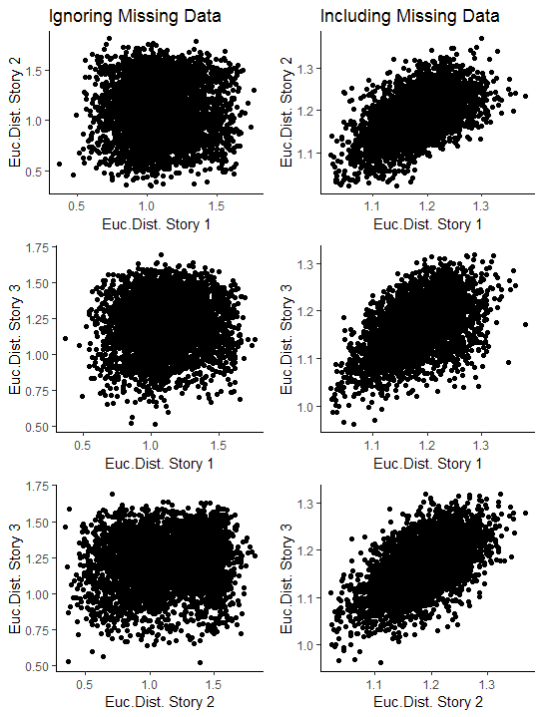
Scatterplots of Euclidean distances between reading behaviors across stories. The figures illustrate how similar each pair of subjects’ gaze durations were in one story, versus how similar they were when reading the other story. Left panels represent data where missing data points were ignored. It is clear that no strong relationship between the reading pattern differences exist. Right panels represent data where missing data points were treated as 0-durations. In this analysis, it becomes clear that subject pairs do show similar reading patterns across stories.

When ignoring missing data points, we found that the distance matrices expressing relative reading behaviors were not consistently correlated across stories (Story 1-Story 2: *r* = .007, *p* = .467; Story 1-Story 3: *r* = .060, *p* = .123; Story 2-Story 3: *r* = .110, *p* = .024) (left panel Fig. 2). However, when taking into consideration the missing data points (as 0-second durations), we observed significant correlations across all story pairs (Story 1-Story 2: *r* = .571, *p* < .001; Story 1-Story 3: *r* = .514, *p* < .001, Story 2-Story 3: *r* = .584, *p* < .001) (right panel Fig. 2). These findings suggest that word skipping might be a reading behavior that is stable across individuals.

One potential concern is that the inclusion of missing data as 0-second durations could introduce artefactually high correlations for participants with rejected data for sections of the story (see Data Preprocessing). In participants with rejected data, not only skipped words were set to a duration of 0 seconds, but also the rejected story sections, which could lead to high correlations if the same sections are missing across participants. Note that in the sample of 102 participants only 2.26% of the total amount of data was rejected (see Data Preprocessing), so overall, data quality was very good. To nevertheless ascertain that our results are not driven by these effects, we repeated the analyses presented above including only participants for whom no data points were rejected at all (*N* = 62, see Data Preprocessing). Scores on the Author Recognition Test for this sub-sample (*M* = 7.35, *SD* = 4.42, range = 1-23) were similar to those in the full sample, suggesting that there was no systematic link between lifetime reading experience and data rejection. Scores on the Story World Absorption Scale for this sub-sample (*M* = 4.31, *SD* = 1.08, range = 1.25-6.71) were also similar to those in the full sample, suggesting that subjective experience of the stories did not systematically influence data rejection.

The results in this more restricted sample resemble those reported above: when ignoring missing data points, we found inconsistent correlations across stories, albeit somewhat stronger ones than when including data sets of participants of which data were removed (Story 1-Story 2: *r* = .082, *p* = .106; Story 1-Story 3: *r* = .121, *p* = .055; Story 2-Story 3: *r* = .187, *p* = .005). When taking into consideration the missing data points (i.e. skipped words) as 0-second durations, we again observed significant correlations across all story pairs (Story 1-Story 2: *r* = .643, *p* < .001; Story 1-Story 3: *r* = .531, *p* < .001, Story 2-Story 3: *r* = .526, *p* < .001), lending support to the idea that word skipping might be a stable, individual-level reading behavior.

Word skipping might be related to individual-level characteristics such as print exposure, attention, or subjective reading experience. We tested these options in a post-hoc analysis using three separate linear regression models in which we estimated whether word skipping (dependent variable) is predicted by individual-level characteristics (independent variable) (package “lme4” version 1.1.17 in R; 30). We used each participant’s score on the Author Recognition Test as a measure of print exposure (i.e. lifetime reading experience), and found that it significantly predicted the number of skipped words (aggregated across all stories) (*B* = 30.84, *SE* = 12.02, *p* = .012 for the full sample; *B* = 40.12, *SE* = 15.15, *p* = .010 for the reduced sample) such that more experienced readers skipped more words.

For the story-level measures of reading experience (attention, story-world absorption), we used a linear mixed effects model with story and participant added as random factors (random intercept). We found no significant relationship between self-rated attention and word skipping (*B* = 3.60, *SE* = 5.42, *p* = .506 for the full sample; *B* = 2.57, *SE* = 7.02, *p* = .714 for the reduced sample), or between story-world absorption and word skipping (*B* = -1.60, *SE* = 6.54, *p* = .928 for the full sample; *B* = .807, *SE* = 8.99, *p* = .928 for the reduced sample). These findings suggest that print exposure, rather than attention or subjective experience, influences word skipping.

## Discussion

We aimed to establish eye movement markers of individual reading styles that are stable across narrative texts in the context of literary reading. We analyzed similarities in word-level gaze durations across participants and across narrative texts, and found that participants’ relative reading behavior is significantly similar across texts when skipped words are taken into consideration. These findings align with previous work that has shown that word skipping is influenced by reader characteristics such as age (31, 32) and reading skill (14, 15). In our analysis, the latter finding is corroborated by the observed correlation between print exposure and word skipping.

Other factors, such as attentional state, might also influence word skipping and blinking, which also leads to—albeit meaningfully—missing data. Indeed, we have recently shown that participants who are more absorbed in a story are more “decoupled” from the text in terms of their reading behavior (27). Missing data, including blinking, and word skipping have also been associated with mind wandering and zoning out during reading (26, 33, 34). However, we found no significant relationship between word skipping and self-reported story-world absorption or attention. It is however possible that a proportion of the missing gaze (i.e. words for which there is no fixation duration available) is associated with one of these individual-level factors. Further research could elucidate these potential relationships. Our results seem to suggest that word skipping should be better understood as a trait as compared to a state-based measure.

It is unlikely that the effects reported here are due to eye tracking issues, such as issues with calibration, given that we find the same results when we include only participants for whom we did not exclude any data. In addition, in between stories, participants left the room and underwent a new calibration session before starting to read the next story. If too many data points were missing (see Data Preprocessing section), stories were excluded, and if too many data points were missing for multiple stories, the participant was excluded. Moreover, the scatterplots shown in the Results section suggest that the correlations are not driven by a few outliers but rather by the distribution across all individuals.

Here, we showed that not only the “tyranny of text” but also a person’s individual reading style influences how gaze is allocated during reading. We established for the first time an eye movement marker of individual reading style: word skipping appears to be a stable individual-level reading behavior across stories.

### Ethics and Conflict of Interest

The authors declare that the contents of the article are in agreement with the ethics described in http://biblio.unibe.ch/portale/elibrary/BOP/jemr/ethics.html and that there is no conflict of interest regarding the publication of this paper.

### Acknowledgements

This research was supported by grant VI.Veni.191G.001 to Myrthe Faber and grant Vidi-276-89-007 to Roel M. Willems from the Netherlands Organization of Scientific Research (NWO).
